# Priapism Followed by Discontinuation of Methadone: A rare Case Report

**Published:** 2015-04

**Authors:** Seyed-Ali Mostafavi, Reza Bidaki

**Affiliations:** 1 Psychiatry and Psychology Research Center, Roozbeh Hospital, Tehran University of Medical Sciences, Tehran, Iran; 2 Research Center of Addiction and Behavioral Sciences, Shahid Sadoughi University of Medical Sciences, Yazd, Iran

**Keywords:** *Priapism*, *Methadone*, *Withdrawal*, *Side effect*, *Fluoxetine*, *Pentoxifylline*, *Case Report*

## Abstract

**Objective:** Priapism is defined by persistent, painful penile erection which occurs without sexual stimulation. Methadone is used as an analgesic and is also used in detoxification and maintenance protocol for opioid dependence treatment. Here we will report a case of a male with priapism after rapid discontinuation doses of methadone.

**Case presentation:** The case was a young married male who referred to a psychiatry clinic due to long-time spontaneous erections. The patient had no history of mental disorders, trauma or sickle cell anemia. He used to smoke opium for five years and used methadone for four years at a dose of 17 cc daily, which he abruptly discontinued. Then he often experienced spontaneous and painful erections without physical or mental stimulation that caused him shame and embarrassment.

**Conclusion:** In this case, chronology indicates that rapid discontinuation of methadone was possibly responsible for the occurrence of priapism. This may have happened due to a compensatory reaction to methadone side effect of erectile dysfunction, followed by its rapid withdrawal.

Priapism is defined as persistent penile erection that is painful and occurs without sexual stimulation (1, 2). Priapism has two types: high-flow (arterial) priapism, in which trauma in the area of penis or pelvic causes an abnormality in arterial flow; and the low-flow (ischemic) type, in which medications, tumors, lumbar disc disease, spinal stenosis, cerebral vascular accidents and hematologic abnormalities (especially sickle cell disease) play a basic role; this type is more common (1, 2). In a study performed by Furtado and et al., the prevalence of priapism was estimated to be about 3.6% in male patients with sickle cell disease (SCD) (3). This rate is even less in the normal population. Ischemic priapism is more likely to cause tissue damage and fibrosis; Veno-occlusion may cause the tissue to become progressively deoxygenated, and this may lead to fibrosis or irreversible tissue damage (4).

This may happen in all age groups, but priapism is more likely to happen in the third and fourth decade of life. It may occur as a very rare side effect of antipsychotic therapy or as a result of multiple medications co-administration with antipsychotics (5).

Methadone (also known as Dolophine) is a synthetic opioid, used as an analgesic and also used in detoxification and maintenance protocol for opioid dependence treatment (6). Few adverse effects are associated with methadone including myosis, hypoventilation, sedation, and constipation. The withdrawal can also induce abstinence syndrome. Hence, the withdrawal should be gradual and its period should be more prolonged compared to other opiates (3% dose reduction per week) (7). Here we report a case of a male with priapism after rapid discontinuation doses of methadone.

## Case Presentation

A young married male, with low educational and socioeconomic status and history of father death was referred to a psychiatry clinic by an urologist for long-time spontaneous erections. Organic and hormonal dysfunctions were rejected by urology examinations, biochemistry tests and patient’s medical history. He did not report usage of any hormone or psychiatry medications in his life except methadone. Sonography of testis and its appendages was normal. Also, the patient had no history of masturbation and bipolar mood disorder. The patient had no history of mental disorders, trauma or sickle cell anemia. Complete blood count activated partial thromboplastic time (aPTT), biochemical parameters and thyroid tests were normal.

He used to smoke opium for five years and used methadone for four years at a dose of 17 cc daily, which he abruptly discontinued. He did not use any additional medications to help stop opium and methadone. The patient tolerated the drug withdrawal symptoms. There was no report of the opioid drug consumption again; and the temptations and common withdrawal symptoms were not reported after one month. However, after 10 days, the patient often experienced spontaneous and painful erections often lasting more than one hour without physical or mental stimulation, and this caused him shame and embarrassment. The pain was not unbearable, so he was not forced to take any pain killers. The patient had a highly increased sexual desire. Sometimes, the erections lasted up to 10 hours. He had no history of spontaneous erections or any medical problems before that.

Prescribed medications included one Medroxyprogesterone acetate daily, Thioridazine 10 mg at night, and Tizanidine 4 mg at night. Nevertheless, no response to treatment was observed during the third session of the follow up. This problem created great distress for him. In the second phase, he took Fluoxetine 10 mg daily and Pentoxifylline 400 mg. Treatment response was started, and Pentoxifylline dose was increased to 400 mg twice a day (i.e., 800 mg daily). Clinical response was completed after three days. We followed him monthly and he hadn’t priapism never after that.

## Discussion

In this case, a painful penile erection that lasted for nearly ten hours was observed six days after the discontinuation of methadone. Chronology indicated that rapid discontinuation of methadone was possibly responsible for the occurrence of priapism in a dose-dependent manner. Methadone is perhaps one of the safest drugs known to use for opium withdrawal with few transit side effects. Priapism has not been reported previously as a methadone side effect and/or as a withdrawal syndrome. 

Most psychoactive drugs act with a "lock and key" mechanism at a receptor level. Atypical antipsychotics such as Clozapine, Olanzapine, Risperidone, Stimulants, Amphetamines and Atomoxetine have antagonist effects on adrenergic alpha-1 receptors, and this is supposed to be the basis for drug-induced priapism (8). In contrast some disorders like irritable bowel syndrome can cause premature ejaculation (9). 

Endorphins are the natural ligand for the opiate receptors, but opium and methadone (opioid agonists) have more affinity to opiate receptors, respectively. Wang and colleagues investigated the long-term effects of methadone on the opioid (δ & μ) and adrenergic (α) receptors in two areas of the rat brain. They found that methadone reduces the cerebral cortical alpha adrenergic receptors (10). Alpha-adrenergic obstruction may cause ischemic priapism (4)

In a study conducted by Tatari F. and et al., Methadone Maintenance Therapy (MMT) was significantly correlated with Erectile Dysfunction(ED); mediators of this association were depression, hypogonadism and Multiple Psychic Traumas. Furthermore, this association was time and dose-dependent. More methadone dose and longer duration of methadone was correlated with more ED (11-13).

In the presented case, spontaneous erections may have happened due to a compensatory reaction to methadone side effect of erectile dysfunction followed by its rapid withdrawal. This response is assumed to be mediated by alpha adrenergic receptors.

## Conclusion

Rapid discontinuation of methadone may cause priapism. This can be an emergency condition and can also induce a psychological distress. Fluoxetine 10 mg daily and Pentoxifylline 400-800 mg daily can be helpful in the treatment of priapism.

## References

[1] Van der Horst C, Stuebinger H, Seif C, Melchior D, Martinez-Portillo FJ, Juenemann KP (2003). Priapism - etiology, pathophysiology and management. International braz j urol : official journal of the Brazilian Society of Urology.

[2] Salonia A, Eardley I, Giuliano F, Hatzichristou D, Moncada I, Vardi Y (2014). European Association of Urology guidelines on priapism. European urology.

[3] Furtado PS, Costa MP, Ribeiro do Prado Valladares F, Oliveira da Silva L, Lordelo M, Lyra I (2012). The prevalence of priapism in children and adolescents with sickle cell disease in Brazil. International journal of hematology.

[4] Kaufman KR, Stern L, Mohebati A, Olsavsky A, Hwang J (2006). Ziprasidone-induced priapism requiring surgical treatment. European psychiatry : the journal of the Association of European Psychiatrists.

[5] Kulmala RV, Lehtonen TA, Tammela TL (1995). Priapism, its incidence and seasonal distribution in Finland. Scandinavian journal of urology and nephrology.

[6] Fredheim OM, Moksnes K, Borchgrevink PC, Kaasa S, Dale O (2008). Clinical pharmacology of methadone for pain. Acta anaesthesiologica Scandinavica.

[7] Senay EC, Dorus W, Goldberg F, Thornton W (1977). Withdrawal from methadone maintenance. Rate of withdrawal and expectation. Archives of general psychiatry.

[8] Eiland LS, Bell EA, Erramouspe J (2014). Priapism Associated With the Use of Stimulant Medications and Atomoxetine for Attention-Deficit/Hyperactivity Disorder in Children. The Annals of pharmacotherapy.

[9] Asad Pour M, ‎Mirzaei V, Bidaki R, Ghorbani AA (2014). The Frequency of Premature Ejaculation in Patients with Irritable Bowel Syndrome. Govaresh.

[10] Wang C, Pasulka P, Perry B, Pizzi WJ, Schnoll SH (1986). Effect of perinatal exposure to methadone on brain opioid and alpha 2-adrenergic receptors. Neurobehavioral toxicology and teratology.

[11] Mohammadi RS, Mahdian M, ‎Bidaki R ( 2014). Essential Thrombocytosis Following Multiple Psychic Traumas. Emergency.

[12] Mostafavi SH, Fazilati M, Vahhabi MR, Omidvarinia Sh (2013). Hepatotoxicity of Dorema aucheri (Bilhar) in albino mice. Arch Iran Med.

[13] Tatari F, Farnia V, Nasiri RF, Najafi F (2010). Trazodone in methandone induced erectile dysfunction. Iranian journal of psychiatry.

